# Gingival health and oral hygiene practices of schoolchildren in the North West Region of Cameroon

**DOI:** 10.1186/s13104-015-1350-2

**Published:** 2015-08-29

**Authors:** Clement Chinedu Azodo, Ashu Michael Agbor

**Affiliations:** Department of Periodontics, University of Benin Teaching Hospital, New Dental Complex, P.M.B. 1111 Ugbowo, Benin City, Edo State 300001 Nigeria; Faculty of Dentistry, Universite des montagnes, Bangangte, Cameroon

**Keywords:** Gingivitis, Oral hygiene, Teeth cleaning

## Abstract

**Background:**

Optimal oral hygiene practices are instrumental to achieving good dental and gingival health. The purpose of this study was to determine the gingival health and oral hygiene practices of schoolchildren in the North West region of Cameroon.

**Methods:**

This cross-sectional survey among 12–13 years old rural and urban schoolchildren in the North West region of Cameroon was conducted between March and November, 2010.

**Results:**

A total of 2295 schoolchildren were interviewed but only 2287 of them had oral examination giving a 99.7 % participation rate. Out of 2287 school children examined, 1676 (73.3 %) had normal gingiva while 26.7 % had gingivitis of varying severity. The gingivitis was found significantly more in rural dwellers (P = 0.001). In terms of the severity of the recorded gingivitis, mild gingivitis constituted 549 (89.9 %), moderate gingivitis 49 (8.0 %) and severe gingivitis 13 (2.1 %). The majority-1929 (85.4 %) of the participants had received instruction on how to care for their teeth and the predominant source of this instruction was from their parents. Irregular teeth cleaning were marked 1137 (49.7 %) among the children. The dominant teeth cleaning materials were toothbrush and toothpaste. The other oral hygiene aid utilized by the participants include dental floss-25 (1.1 %), stick-759 (33.6 %), dental floss-25 (1.1 %) and other unorthodox agents. The reasons for teeth cleaning among the participants in descending order were to make the teeth clean, to prevent halitosis, to make teeth stronger, to prevent pain and dental diseases.

**Conclusion:**

The prevalence of gingivitis among Cameroonian schoolchildren in the Northwest region was 26.7 % with majority being of mild gingivitis category. Parents, dental professionals and teachers were the main sources of instruction on oral care.

## Background

Oral hygiene positively affects mastication, eating, swallowing, speech, facial aesthetics and social interaction thereby cumulating in improved quality of life. Teeth cleaning is an established oral hygiene modality which entails the removal of dental plaque, stains and other deposits from teeth to prevent mainly dental caries and periodontal diseases.

Historically, chewing sticks (miswak), baking soda, chalk, potash, alum and charcoal have been used for teeth cleaning but in modern times, tooth brushing and toothpaste is the most commonly used method. The choice of toothbrush is mostly due to the perceived efficacy while the choice of chewing stick is hinged on availability, religion and traditional beliefs [1, 2]. Optimal oral hygiene practices are instrumental to achieving good dental and gingival health. However, optimal oral hygiene practices are hindered by poverty, ignorance, misinformation, erroneous beliefs, cultural and religious practices. The dependence of gingival health on oral hygiene practices will therefore be invariably affected by these impediments. Gingivitis which is a dominant gingival disease, is common in childhood and may progress to periodontitis, if left untreated. Bacterial plaque and it accumulation is strongly associated with gingivitis [3–5].

Hence daily home based mechanical plaque removal is critical for the maintenance of gingival health and when efficiently performed, leads to remission of gingivitis [6]. Adequate information on oral health from informed source and positive attitude are expected to motivate effective oral self-care behaviour with resultant periodontal health. Significant improvements in plaque removal following good brushing instructions regardless of the toothbrush design used among children has been reported [7]. This emphasized the importance of teaching children, the optimal oral hygiene to facilitate the prevention of gingivitis. The gingival index by Loe and Silness [8] is the accepted and preferred method of assessing gingival health because it helps in determining the prevalence and severity of gingival disease. There is dearth of information on the oral hygiene practices and gingival health among schoolchildren of developing countries. The purpose of the study was to determine the gingival health and oral hygiene practices of schoolchildren in the North West region of Cameroon.

## Methods

### Ethical consideration

The protocol for this study was reviewed and approval granted by from the Ministry of Basic Education and Public Health in the North West region of Cameroon. Assent and consent was obtained from children and parents or guardian of the children, respectively.

### Study design/study setting

This baseline cross-sectional survey was conducted among rural and urban schoolchildren in the North West region of Cameroon between March and November, 2010. The Northwest region is the one of the ten regions that make up Cameroon. It is administratively divided into seven divisions namely *Boyo, Bui, Donga*-*Mantung, Menchum, Mezam, Momo* and *Ngo*-*ketunjia.* Northwest region is third most populated region in Cameroon with an estimated population 1.8 million in 2010.

### Sampling

Multistage sampling techniques was employed in this study. In the first stage, the seven divisions that make up the North West region were arranged in alphabetical order. Two yes and five no responses were keep in a box and seven patients attending the Dental Department, Nkwen Baptist Health Centre, Bamenda, Cameroon assisted in picking the responses. The first picked response corresponded to the first division in alphabetical order while the last picked response corresponded to the last division in alphabetical order. At the end of the exercise, Bui and Mezam divisions were selected.

At the second level of sampling, convenience sampling technique was employed in selecting the schools in the divisional headquarters so as to get a large population and to facilitate the inclusion of rural and urban schools in the study. Bamenda and Kumbo which are the divisional headquarters of Mezam and Bui were selected. In the two locations, the urban and the rural schools were included.

In each school, all the 12–13 years old children were included to make it a large population study. The children that did not assent to the study, those that could not provide parental or guardian permission and those that reported suffering from chronic systemic diseases were excluded. The systematic sampling technique was used in selecting the participants using school registers.

### Data collection tool/procedure

The data collection was done through interviewer- administered questionnaire and clinical oral examination. Interviewer-administered questionnaire elicited information on frequency and reasons for tooth cleaning, utilized tooth cleaning aids and source of information on oral self-care. This questionnaire was pre-tested among school children from the non-selected schools in the region. Oral examination of the selected schoolchildren was carried out under natural light using examination gloves, disposable probes and sterile disposable mouth mirrors. Dental therapists who were trained and calibrated before the start of survey recorded the findings. The status of gingival health was examined on the buccal, lingual mesial and distal teeth with gingival index with scoring as follows: 1, mild inflammation; 2, moderate inflammation; 3, severe inflammation. The score for each individual was gotten by adding all the gingival score and dividing it by the total number of teeth multiplied by four (4). The status of gingival health was subsequently categorized based on the gingival score as 0, normal gingival; 0.1–1, mild gingivitis; 1.1–2.0, moderate gingivitis; 2.1–3.0, severe gingivitis.

### Data analysis

Data analysis was done using the Statistical Package for the Social Science (SPSS) version 17.0. Test of association was done using Chi Square statistics and P < 0.05 was considered as statistical significance.

## Results

A total of 2295 schoolchildren were interviewed but only 2287 of them had oral examination giving a 99.7 % participation rate. We found no any differences in the characteristics of the participants and non-participants both at the pretest and the final study stages. Out of 2287 schoolchildren examined, 1676 (73.3 %) had normal gingiva while 26.7 % had gingivitis of varying severity. Males, rural dwellers and irregular teeth cleaners had more gingivitis. However it was only among the rural dwellers that the prevalence was significantly higher than the urban dwellers (P = 0.001) (Table 1). In terms of severity of recorded gingivitis, mild gingivitis constituted 549 (89.9 %), moderate gingivitis 49 (8.0 %) and severity gingivitis 13 (2.1 %). There was no significant association between gender, location, regularity of teeth cleaning and the severity of gingivitis (Table 2). Irregular teeth cleaning among the participants were marked 1137 (49.7 %) with this being higher among females and rural dwellers than their counterparts (P = 0.001) (Table 3). Toothbrush and toothpaste were the most commonly teeth cleaning method as 2138 (94.5 %) had personal toothbrush and 63 (2.8 %) shared toothbrush with other members of the family. The other oral hygiene aids utilized by the participants include chewing stick-759 (33.6 %), dental floss-25 (1.1 %) and other unorthodox agents (Table 4). The reasons for tooth cleaning among the participants were to make the teeth clean, prevent mouth odour, make teeth stronger, prevent pain and dental diseases (Table 5). The majority 1929 (85.4 %) of the participants have received instruction on how to care for their teeth from with their parents 1227 (53.7 %) being the predominant source (Table 6).

**Table 1:** Prevalence of gingivitis among the participants

Gingivitis	Gender		Location		Teeth cleaning		
	Male, n (%)	Female, n (%)	Urban, n (%)	Rural, n (%)	Regular, n (%)	Irregular, n (%)	Total, n (%)
Absent	817 (71.7)	859 (74.8)	902 (76.1)	774 (70.2)	855 (74.3)	821 (72.2)	1676 (73.3)
Present	322 (28.3)	289 (25.2)	283 (23.9)	328 (29.8)	295 (25.7)	316 (27.8)	611 (26.7)
Total	1139	1148	1102	1185	1150	1137	2287
P-value	0.094		0.001		0.247

**Table 2:** Severity of gingivitis among the participants

Gingivitis	Gender		Location		Teeth cleaning		
	Male, n (%)	Female, n (%)	Urban, n (%)	Rural, n (%)	Regular, n (%)	Irregular, n (%)	Total, n (%)
Mild	284 (88.2)	265 (91.7)	255 (90.1)	294 (89.6)	270 (91.5)	279 (88.3)	549 (89.9)
Moderate	30 (9.3)	19 (6.6)	25 (8.8)	24 (7.3)	19 (6.5)	30 (9.5)	49 (8.0)
Severe	8 (2.5)	5 (1.7)	3 (1.1)	10 (3.1)	6 (2.0)	7 (2.2)	13 (2.1)
Total	322	289	283	328	295	316	611
P-value	0.360		0.194		0.373

**Table 3:** Frequency of tooth cleaning among the participants

Teeth cleaning	Gender		Location		
	Male, n (%)	Female, n (%)	Urban, n (%)	Rural, n (%)	Total, n (%)
Regular	596 (52.3)	554 (48.3)	665 (56.1)	485 (44.0)	1150 (50.3)
Irregular	543 (47.7)	594 (51.7)	520 (43.9)	617 (56.0)	1137 (49.7)
Total	1139	1148	1185	1102	2287
P-value	0.052		0.001

**Table 4:** Utilization of oral hygiene aid among the participants

Oral hygiene aid	Frequency (no.)	Percent (%)
Personal toothbrush	2138	94.5
Communal toothbrush	63	2.8
Thread/dental floss	25	1.1
Chewing stick	759	33.6
Finger	467	20.6
Charcoal	457	20.2
Salt	1135	50.2
Soap	423	18.7

More than one teeth cleaning aid by participants

**Table 5:** Reasons for tooth cleaning among the participants

Reasons	Frequency (no.)	Percent (%)
To keep my teeth clean	1704	74.5
To prevent mouth odour	722	31.6
To make my teeth strong	301	13.2
To prevent dental diseases	117	5.1
To avoid pain	71	3.1
To prevent tooth discolouration	32	1.4

More than one reason for teeth cleaning by participants

**Table 6:** Sources of information on care of the teeth among the participants

Source	Frequency (no.)	Percent (%)
Parents	1227	53.7
Dental professionals	563	24.6
Teachers	377	16.5
Media	55	2.4
Other health workers	44	1.9
Friends	35	1.5
Others	18	0.8

## Discussion

Gingivitis is reversible and rarely progresses to periodontitis in childhood except in special circumstances like aggressive periodontitis and periodontitis associated with systemic diseases. In this study, 26.7 % had gingivitis of varying severity. This is lower than 71.1 and 46.2 % reported in suburban Nigerian school children [9] and rural Cambodian [4], respectively. The low prevalence of gingivitis may be explained by the high ownership of toothbrush in this study as gingival disease in schoolchildren is linked to non-toothbrush ownership [4]. Males had more gingivitis than females in this study. Gender variation in gingival index has been noted among Nigerian, Sudan and Jordan schoolchildren [9–11]. Although irregular teeth cleaning was higher in females than males, the low efficiency of teeth cleaning even with toothbrush in developing countries due to poor attention given to oral self-care by the male folk right from young age may be the explanation [12].

The gingivitis recorded was significantly higher among schoolchildren in rural area than those in urban area. Although with a different index of assessment, this study finding was similar to the report of rural–urban difference in Morogoro District of the Republic of Tanzania [13]. There also exist reports of worse periodontal scores in rural dwellers compared urban dwellers [14, 15]. Reports of variation in oral health knowledge, attitude and practices in rural and urban dwellers with poorer parameters in rural dwellers may be the explanation for rural–urban periodontal disease gradient [16]. The irregular teeth cleaning which was significantly higher among rural dwellers than the urban dwellers in this study may also be a contributory explanation.

The severity pattern of gingivitis reported in this study was in tandem with a previous report in Nigerian schoolchildren [9]. The fact that the majority of children with gingivitis were of mild severity means that the resolution can occur with adherence to proper oral hygiene instruction among the affected schoolchildren. It is therefore important to instruct schoolchildren in excellent oral hygiene in order to prevent gingivitis and its further deterioration. This emphasizes that dentists should endeavor to give information and training on regular plaque removal on any ethically acceptable clinical and non-clinical encounter [6]. However, parents constituted the predominant source of oral hygiene information among the schoolchildren in this study which is similar to 74 % of the northern Lebanon children who learnt how to brush their teeth from their mother [17]. The known low oral health manpower population ratio in developing countries may explain why dentist was the lesser source of oral health information than parents. Based on this barrier, the provision of intensive advice and supervision concerning oral hygiene practices through public school healthcare is therefore a viable alternative. The limited oral hygiene knowledge of the parents who constituted the predominant source of oral hygiene information may explain the marked irregular teeth cleaning among the schoolchildren. This irregular teeth cleaning was higher than values reported in Sudan [11] and Kuwait [18] schoolchildren. This marked irregular teeth cleaning was higher among females than males, which contrasted with the established norm of females indulging more in regular teeth cleaning than males [18, 19]. The influence of parents on oral health practices of children though not assessed in this study is a suggested explanation for differing from norm gender difference in teeth cleaning regularity [20].

In this study, toothbrush was the most commonly teeth cleaning method as 94.5 % had personal toothbrush and 2.8 % shared toothbrush with other members of the family. Although the sharing of toothbrush is low, it needs to be discouraged because it can serve as mode of transmission of infection like herpes infection. The use of toothbrush for teeth cleaning in this study was comparable to reports among Nigerian adolescents (96.3 %) [21], Sudan (93.1 %) [11] and Kenyan schoolchildren (92.0 %) [19]. However, it was higher than 47.1 and 75 % reported in children in Dhaka [22] and North Lebanon [17]. This high use of toothbrush and toothpaste may be due to their perceived efficacy in teeth cleaning. This also signifies its adoption as the most effective method to maintain healthy conditions for teeth and gingiva even in developing countries as a welcome development [1]. The use of chewing stick is still very popular because of availability, lower cost than the toothbrush and paste [1, 2]. Chewing stick was a prominent oral hygiene aid while use of dental floss was unpopular in this study which collaborated with findings of studies among residents of many developing countries [16, 22, 23]. Use of charcoal, salt, soap and other unorthodox agents for teeth cleaning in this study appear to exist in many other developing countries especially in the rural settings. There exists report of charcoal and ash use in rural areas of the Ilala district in Tanzania [12, 22, 24]. Cleaning of teeth helps to remove plaque and stains thereby invariably preventing oral diseases and maintaining aesthetics. It can therefore be stated that the motives for teeth cleaning is either for disease prevention or aesthetic reason. In this study, the reasons for teeth cleaning were to make the teeth clean, prevent mouth odour, make teeth stronger, prevent pain and dental diseases. This indicates that aesthetic reasons took precedence over preventive dental health reasons and this is similar to reports in Sweden [25], England [26] and South Africa [27]. The marked irregularity in teeth cleaning in the schoolchildren in this study explained the favorability of aesthetic reason as it is known that the main motive of teeth cleaning for those who clean their teeth less frequently is cosmetic reasons, that is, having their teeth, look and feel good and having fresh breath smells, in contrast to the avoidance of toothache and false teeth, which are health reasons [28].

The questionnaire used in this study was self-developed for the study and pretested so does not have a long established history of validity. The findings of this study should be interpreted with caution because self-reported oral health behavior may not necessarily reflect actual behaviors.

## Conclusion

In conclusion, the prevalence of gingivitis among Cameroonian school children in Northwest province was 26.7 % with majority being of mild gingivitis category. Irregularity in teeth cleaning was marked and the reasons for tooth cleaning were more aesthetic than preventive reason. Dental professionals were not the leading source of instruction on oral self-care.
